# OliveAtlas: A Gene Expression Atlas Tool for *Olea europaea*

**DOI:** 10.3390/plants12061274

**Published:** 2023-03-10

**Authors:** Amanda Bullones, Antonio Jesús Castro, Elena Lima-Cabello, Juan de Dios Alché, Francisco Luque, Manuel Gonzalo Claros, Noe Fernandez-Pozo

**Affiliations:** 1 Institute for Mediterranean and Subtropical Horticulture “La Mayora” (IHSM-CSIC-UMA), 29010 Málaga, Spain; 2 Department of Biochemistry and Molecular Biology, Universidad de Málaga (UMA), 29010 Málaga, Spain; 3 Plant Reproductive Biology and Advanced Imaging Laboratory (BReMAP), Estación Experimental del Zaidín (CSIC), 18008 Granada, Spain; 4 Instituto Universitario de Investigación en Olivar y Aceites de Oliva, Departamento de Biología Experimental, Universidad de Jaén (UJA), 23071 Jaén, Spain; 5 Institute of Biomedical Research in Málaga (IBIMA), IBIMA-RARE, 29010 Málaga, Spain; 6 CIBER de Enfermedades Raras (CIBERER), 29071 Málaga, Spain

**Keywords:** *Olea europaea*, RNA-seq, bioinformatics, gene expression, abiotic stress, *Verticillium*, Picual, pollen germination

## Abstract

The olive (*Olea europaea* L.) is an ancient crop of great importance in the Mediterranean basin due to the production of olive oil and table olives, which are important sources of fat and have benefits for human health. This crop is expanding and increasing its production worldwide and five olive genomes have recently been sequenced, representing a wild olive and important cultivars in terms of olive oil production, intensive agriculture, and adaptation to the East Asian climate. However, few bioinformatic and genomic resources are available to assist olive research and breeding, and there are no platforms to query olive gene expression data. Here, we present OliveAtlas, an interactive gene expression atlas for olive with multiple bioinformatics tools and visualization methods, enabling multiple gene comparison, replicate inspection, gene set enrichment, and data downloading. It contains 70 RNA-seq experiments, organized in 10 data sets representing the main olive plant organs, the pollen germination and pollen tube elongation process, and the response to a collection of biotic and abiotic stresses, among other experimental conditions. OliveAtlas is a web tool based on easyGDB with expression data based on the ‘Picual’ genome reference and gene annotation.

## 1. Introduction

The olive (*Olea europaea* L.) is a crop of great social and economic importance due to the production of oil, which is the main fat source in Mediterranean countries and has benefits for human health [1]. In addition, some cultivars are grown for the quality of their fruits for consumption as table olives, and other parts of the plant are of utility for human use, such as its wood for firewood, and its seeds, which have been shown to have great potential as a source of edible oil, nutraceuticals, and proteins or meal serving as feed supplements [2]. Currently, most of the olives are cultivated in the Mediterranean basin, with Spain, Italy, and Greece as the top producers of olive oil worldwide. However, this crop is expanding and increasing its production in other regions of the world such as the United States, China, Australia, and South America. Its cultivation occupies more than 11 million hectares worldwide, of which 98% are located in the Mediterranean basin [3] and 21% of them in Spain, where the olive is a strategic crop for the agri-food industry.

It is believed that the domestication of the olive occurred at least 7000 years ago in the Middle East from wild olive progenitors [4,5] and it spread from the east to the west of the Mediterranean basin with human migrations [6]. Olives might have been exploited for at least 19,000 years [7] since the use of wild olives has been described in different parts of the Mediterranean basin during the Neolithic. It is estimated that there are more than 2000 olive varieties worldwide, which are clonally propagated by cuttings or grafts [6].

In 2016, the genome of the cultivated olive (*Olea europaea* L. subsp. *europaea* var. *sativa*) was sequenced for the first time, using an individual of the cultivar Farga [8]. Recently, this genome assembly was improved by anchoring it to a genetic map [9]. In 2017, the genome of the wild olive or oleaster (*O. europaea* L. subsp. *europaea* var. *sylvestris*) from a Turkey-located individual, was also published [10], but a recent work suggested it could have been a feral individual that may have been misidentified [9]. In addition, the genome sequences of two widely cultivated olive cultivars, ‘Picual’ and ‘Arbequina’ became available in 2020 and 2021, respectively [11,12]. ‘Picual’ is the most cultivated variety in the world, responsible for approximately 25% of the production of olive oil worldwide, and ‘Arbequina’ is of special interest because of its lower vigor, which allows mechanized harvesting and dense planting for intensive agriculture. Moreover, in 2022, the genomes of two olive trees of the subspecies *cuspidata* were sequenced because of their usability as rootstock with better disease resistance, and adaptation to East Asian climate [13,14].

A large increase in knowledge has been achieved thanks to the five olive genomes recently sequenced. However, there are still not many bioinformatic and genomic resources to assist olive research and breeding. Despite gene expression data being crucial for functional genomics, to know where and when genes are expressed, the only published platform for gene expression query for olive is ReprOlive [15], which was based on Roche 454 sequencing and is no longer accessible. Moreover, many olive gene expression studies published are based on de novo transcriptomes, which do not share a common gene reference and annotations. Here, we present OliveAtlas, an interactive gene expression atlas for olive where expression data were analyzed using the cultivar Picual genome and its gene model annotation as references. OliveAtlas is available at https://www.oliveatlas.uma.es/ (accessed on 24 February 2023).

## 2. Results

### 2.1. Datasets Available in OliveAtlas

This first version of OliveAtlas includes a selection of the most relevant published RNA-seq experiments from the Picual cultivar, which were reanalyzed based on the ‘Picual’ genome sequence and gene annotation. Additionally, the only two olive datasets based on genomic references found in NCBI GEO [16] were added as an example of how simple it is to add new datasets to OliveAtlas, now that several genomic references are available: ‘Picual’, ‘ Farga’, wild olive, and the subspecies *cuspidata*. The datasets found in NCBI GEO include root experiments of many olive varieties with different tolerance to *Verticillium dahliae* infection and expression data of the ‘Souri’ variety exposed to drought conditions. (Table 1). The ‘Picual’ datasets available in OliveAtlas include experiments already published, with a representation of the organs of the olive plant [17], and a collection of biotic and abiotic stresses to understand the response of olive leaves and roots to cold, wound, and infection by *V. dahliae* [18]. Additionally, experiments of pollen in vitro germination, which include a time series of mature rehydrated pollen and germinated pollen at 1, 3, and 6 h of culture [19], and gene expression data from the whole seed, as well as from the testa plus endosperm and embryo tissues separately, were included. A total of 70 different experiments were organized in 10 datasets in OliveAtlas (Table 1). The experiments were included as they were organized in their respective publications, and in addition, the stress experiments were separated into new datasets by each stress type (cold, wound, and *V. dahliae* infection). An additional dataset was included to collect all available tissues in control conditions (roots from adult individuals, roots from 4-month-old plants, stems, meristems, leaves from adult individuals, leaves from 4-month-old plants, whole flowers just after anthesis, mature pollen grains, fruits at veraison stage, and mature seeds).

**Figure 1:**
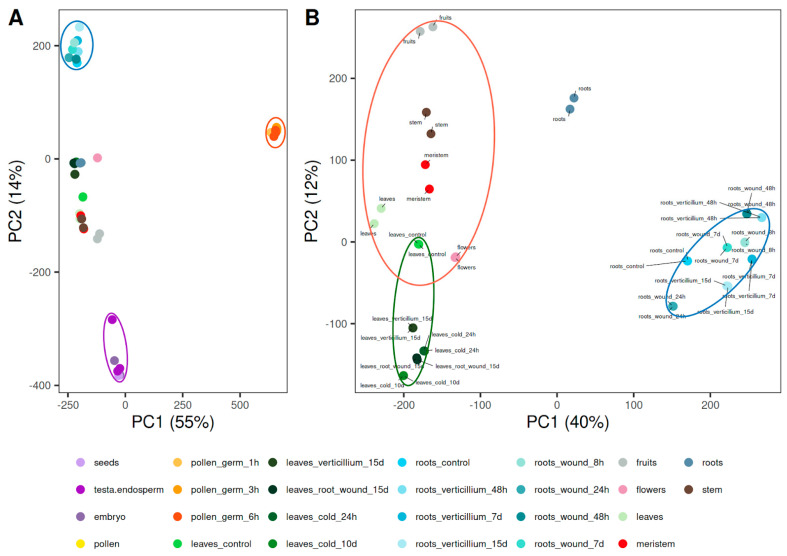
Principal component analysis of OliveAtlas ‘Picual’ samples. (**A**) PCA of all samples. (**B**) Detailed distribution of samples excluding seeds and pollen datasets. Orange ellipse groups pollen samples, purple ellipse groups seed samples, blue ellipse groups stress experiments conducted in roots, red ellipse groups plant organs under control conditions and green ellipse groups leaf experiments under stress conditions.

A Principal Component Analysis (PCA) was done with the replicates of the ‘Picual’ experiments (Figure 1), showing the pollen samples (in orange) to be the most different ones to the rest, followed by the samples from the seed (in purple), and the root stress experiments (in blue), which are also separated from the rest but only in the second component (Figure 1A). Another PCA without the pollen and seed samples showed a clear separation of experiments of root stresses, leaf stresses, and the remaining tissues in control conditions (Figure 1B). Replicates from each experiment clustered close to each other. Leaves under control conditions from “Picual stresses” [18] and from “Picual plant organs” [17] datasets are also close to each other. However, root samples under control conditions from 10-year-old individuals (from the “Picual plant organs” dataset) are separated from the rest of the tissues under control conditions and the other root datasets in the PCA (Figure 1B). Roots under control conditions from the “Picual stresses” dataset were extracted from 4-month-old plants and seem to be much closer to the rest of the 4-month-old root samples under stress conditions than to the 10-year-old root samples under control conditions.

### 2.2. Tissue/Organ Specificity of ‘Picual’ Genes

From the 81,484 total genes annotated in the ‘Picual’ genome [12], 56,348 genes (69.15%) showed expression (≥2 TPMs, Transcripts Per Million) in at least one of the tissues of *O. europaea* cv. Picual included in OliveAtlas. Up to 25,136 genes (30.85%), showed no expression or very low expression [8319 genes (10.21%) with 0 TPMs and 16,817 genes (20.64%) with TPM values between 0 and 2]. The average length and exon number of expressed genes are higher than those from the low or not expressed genes, with average values of 1719.50 bp and 5.96 exons in expressed genes, and 836.49 and 3.52 exons in low or not expressed genes, respectively. The total number of expressed genes and genes specifically expressed in each tissue under control conditions are summarized in Table 2, where roots and meristems have the highest number of expressed genes, while pollen has the lowest number of expressed genes. On the other hand, pollen grains, roots, and flowers show the highest number and percentage of specific genes, while stems expressed the lowest number and percentage of specific genes. Specific genes of all tissues under control conditions are available in Table S1.

**Figure 2:**
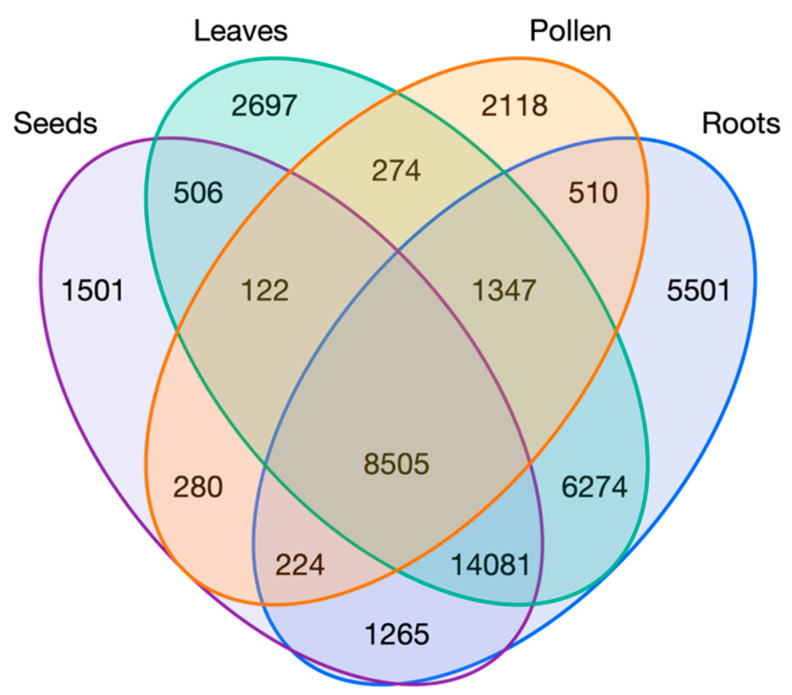
Venn diagram of expressed genes in a simplified tissue representation of the ‘Picual’ experiments under control conditions.

Pollen and seeds, as the most different tissues in the PCA (Figure 1A), together with roots, as the tissue with the highest number of specific genes, and leaves as representative of photosynthetic tissue, all under control conditions, were compared in a Venn diagram (Figure 2). The group with the highest number of genes (14,081) corresponded to the union of all tissues but pollen, which, as shown before, was the most distant from the rest of the tissues in the PCA and had the highest percentage of specific genes. Then, 8505 genes were found in all tissues, and 6274 were shared by roots and leaves but were absent in pollen and seeds, which were separated from the rest of the tissues in the PCA (Figure 1A). The reduction of the number of tissues compared in the Venn diagram showed a higher number of specific genes in comparison with the analysis including all tissues presented in Table 2. This result was expected since, for example, leaf specific genes increased in 2089 when there were no other photosynthetic tissues represented. Specific genes from the comparison of leaves, roots, pollen, and seeds under control conditions are available in Table S2.

### 2.3. Functional Enrichment Analysis of ‘Picual’ Genes

**Figure 3:**
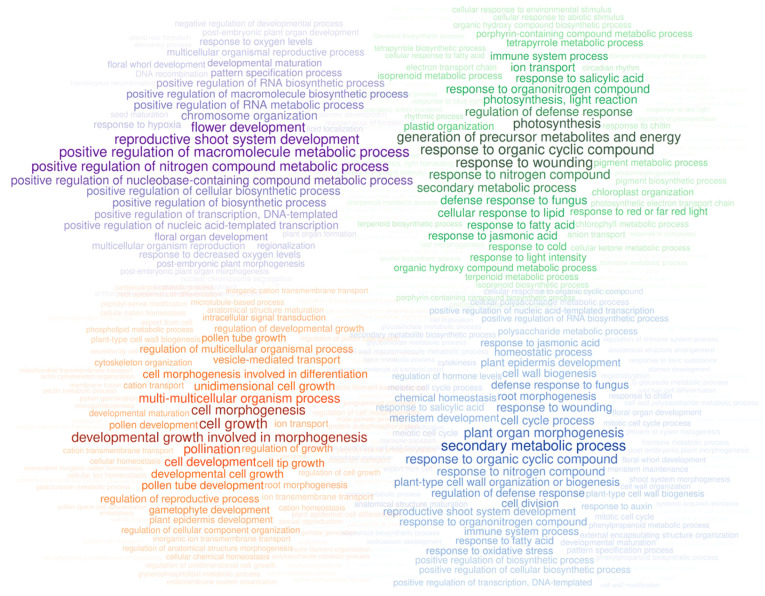
Enriched biological processes in specific genes of seeds (**top-left**, in purple), leaves (**top-right**, in green), pollen (**bottom-left**, in orange), and roots (**bottom-right**, in blue). Word size and color opacity represent the frequency of the terms found in the enrichment analysis.

The functions corresponding to the genes specifically expressed in seeds, pollen, leaves, and roots, under control conditions, were characterized by functional enrichment analysis of the biological processes associated with those genes (Figure 3). In the seeds (including samples of the embryo, and endosperm with testa tissues, as well as one replicate of the whole seed), it was observed a positive regulation of many metabolic and biosynthetic processes, and other interesting terms related to development, chromosome organization, and organ morphogenesis. In leaves, control samples from 4-month-old olive plantlets and 10-year-old trees were included. In this organ, there are terms mostly related to photosynthesis and energy but also related to defense responses against biotic and abiotic stresses and response to several endogenous stimuli (e.g., phytohormones). Pollen samples (represented by rehydrated mature pollen and germinated pollen) showed biological processes related to pollination, pollen tube tip growth, cell morphogenesis, and vesicle-mediated and ion transport. Finally, root samples (comprehended by samples of 4-month-old plantlets and 10-year-old adult individuals under control conditions), included terms related to morphogenesis, secondary metabolic processes, and defense responses. Enrichment results are available in Table S3.

### 2.4. Analysis of ‘Picual’ Constitutive Genes

Genes that are constitutively expressed in most tissues and experimental conditions and that are required for the maintenance of basic cell functions, also known as housekeeping genes, are useful as references for normalizing gene expression in RT-PCR experiments. Genes from all ‘Picual’ data sets were analyzed to identify genes expressed with coefficients of variation less than 0.2. Seven genes with a low coefficient of variance were found (Table 3).

### 2.5. OliveAtlas Navigation and Tools

OliveAtlas is an open-access gene expression atlas tool based on easyGDB [22] that provides multiple user-friendly tools and interactive visualization methods to explore olive gene expression data. The OliveAtlas website can be accessed anonymously at https://www.oliveatlas.uma.es/ (accessed on 24 February 2023) with no account or login required (Figure S1). Additionally, information about the research institutions involved in the development of the tool and the founding source can be found in the About Us section (Figure S2), where it is also possible to contact the administrators to request the addition of new datasets or suggest the development of further tools and features. Moreover, the help section in the about tab of the toolbar provides detailed instructions on how to use the OliveAtlas tools.

#### 2.5.1. Expression Data File Downloading

The Downloads section contains compressed files with the expression data of all replicates for each one of the datasets in tab-delimited text format, as well as the unique transcripts of ReprOlive [15] in FASTA format and their annotations in tab-delimited text files (Figure S3).

#### 2.5.2. Expression Atlas Tools

The OliveAtlas “Expression Atlas” toolbar menu provides access to two tools, the Gene Expression Viewer and the Expression Comparator, and to the dataset information. The Datasets section describes the experimental information of the datasets, including related publications, links to source data, and images of the experiments when available (Figure S4). The Gene Expression Viewer is the main tool of OliveAtlas. Here, it is possible to choose between all the datasets mentioned in Table 1 and provide a list of genes to compare their expression values in multiple visualization tools. On the other hand, the Expression Comparator allows the combination of samples from any dataset to visualize their gene expression values, with the possibility of calculating fold change values and log ratios relative to one or more genes, which for example, could be taken from the least variable genes identified in Table 3.

The input form of both tools assists users to autocomplete gene names to avoid spelling mistakes and add them to the input gene list (Figure S5), or the genes used for fold change calculation. The results of the Gene Expression Viewer describe the experimental conditions of the selected dataset and provide five visualization methods: (1) Lines, (2) Expression cards, (3) Heatmap, (4) Replicates, and (5) Average values.

**Figure 4:**
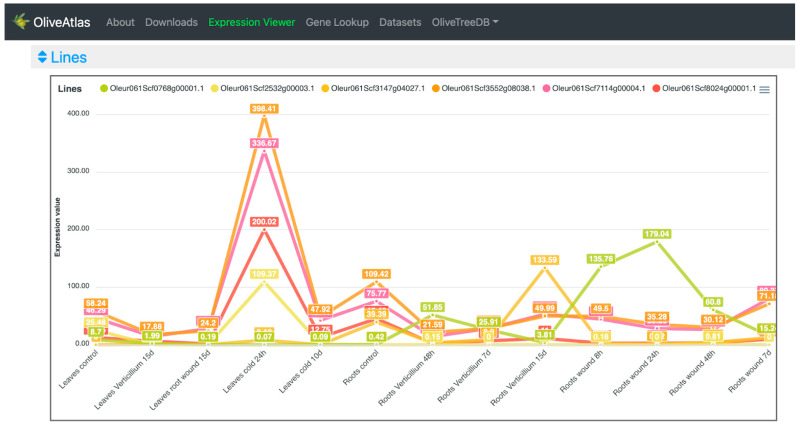
OliveAtlas “Expression viewer” lines plot. Example of six genes described in [18], showing different responses to several stresses.

**Figure 5:**
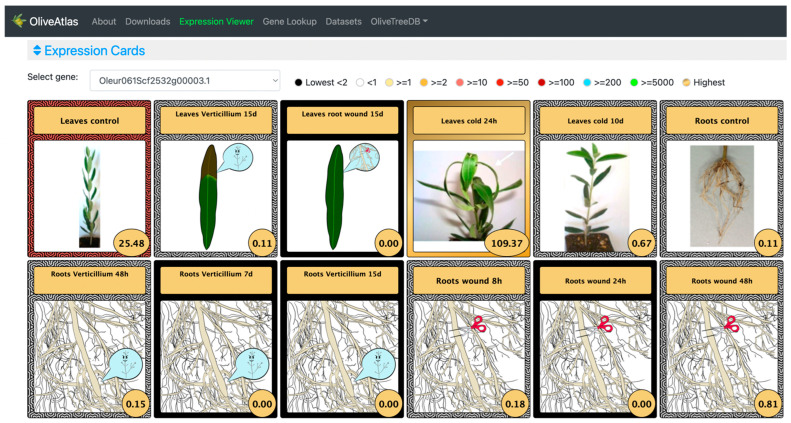
Expression cards in the “Expression viewer” of OliveAtlas. The cards show different colors for different expression value ranges defined in the legend. The highest and lowest values are highlighted in a golden or black card respectively. The sample *Roots wound 7d*, with expression value 0, was not included for better visualization.

**Figure 6:**
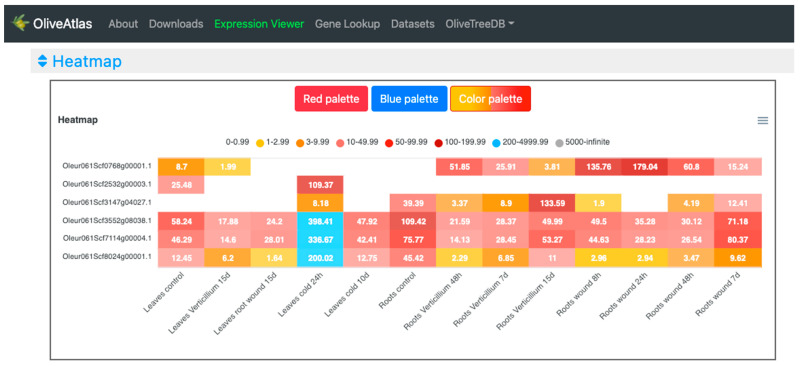
Heatmap visualization of the “Expression viewer” in OliveAtlas. Expression data are displayed grouped by color in several ranges of expression. Three different color palettes are available.

**Figure 7:**
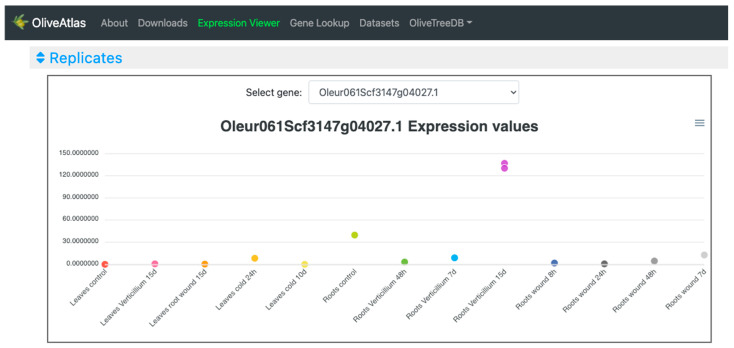
Expression values of replicates of an example gene in the “Expression viewer” tool of OliveAtlas.

**Figure 8:**
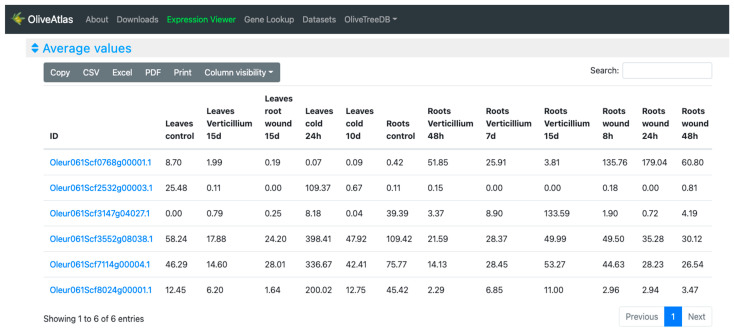
Average values table in the “Expression viewer” tool of OliveAtlas. More experimental data are available when scrolling to the right of the table.

In the “Lines” visualization, the expression of all genes can be compared (Figure 4). In some cases, the expression values and lines of some genes hide the information of others, but the tool is very interactive, and it is possible to place the cursor over the gene names of the legend on top of the graph to highlight the selected gene. It is also possible to click on these gene names to show and hide the data in the graph, to easily compare the desired genes. On the other hand, moving the cursor over the data points will show the values of all genes in the experimental sample selected. Additionally, the graph can be downloaded in different formats (SVG, CSV, or PNG) to save an image of the plot or its data. In the “Expression cards”, we can select each one of the genes to see their expression values together with pictures showing their phenotype in the experimental conditions or drawings representing the tissue (Figure 5). The highest value is highlighted on a golden card and the lowest ones are on black cards. The “Heatmap” option (Figure 6), allows a simultaneous comparison of all genes and experimental conditions using a color scale that separates expression values in different ranges. Moving the cursor over the different color ranges in the legend will highlight the expression values within that range. The “Replicates” visualization (Figure 7) allows the inspection of the replicates of the selected gene. Finally, the “Average values” section (Figure 8) provides a table with the values of each gene in each experiment. This table can be downloaded in several formats (CSV, Excel, PDF), can be copied to the clipboard, filtering out unwanted columns, and searching within the information in the table. The gene names in the table are linked to the gene annotation page in OliveTreeDB, where it is possible to find sequences and annotations of the genes together with the visualization of the gene in the genome browser.

The Expression Comparator output includes the Lines, Heatmap, Replicates, and Average values as in the Gene Expression Viewer but it can show fold change or log ratio values (Figure S6).

#### 2.5.3. Gene Lookup and Gene Set Enrichment Tool

The gene lookup tool (Figure S7) allows the conversion of a list of genes between the different gene sets from the cv. Picual and the sequenced genotypes cv. Farga, cv. Arbequina, wild olive, Arabidopsis, and the transcriptomes from ReprOlive [15]. That way, it is possible to get the most similar gene IDs from the ‘Picual’ gene annotation and use these ‘Picual’ identifiers to query any dataset in OliveAtlas and the OliveTreeDB tools [12] (https://genomaolivar.dipujaen.es/ (accessed on 24 February 2023)). Conversely, a list of ‘Picual’ identifiers can be rapidly converted to any of the other genome references to use external bioinformatics tools and resources, or to identify putative orthologs mentioned in the literature in other olive cultivars or Arabidopsis. ReprOlive includes datasets based on transcriptomes of pollen, pistil, seed, and vegetative tissues from the cv. Picual. The results of the Gene lookup tool can be copied to the clipboard, printed, or downloaded in different formats such as CSV, Excel, or PDF.

The Gene Set Enrichment tool (Figure S8) uses the gene lookup tool code to convert the gene identifiers to the most similar genes from Arabidopsis, ‘Farga’, or wild olive to perform gene set enrichment of Gene Ontology terms in g:Profiler [23].

#### 2.5.4. OliveTreeDB Data and Tools

OliveTreeDB is the main reference genomic portal of olive cv. Picual, including genome data, annotations, and tools to access the information [12]. The results from the OliveAtlas “Expression Viewer” tool, described below, are linked to the OliveTreeDB gene annotation pages. Additionally, the OliveAtlas toolbar menu provides direct links to the resources and tools available at OliveTreeDB (Figure S9): (1) Downloads: where it is possible to get gene and genome annotation and sequence files; (2) BLAST: to search sequences by similarity against cv. Picual sequences (genome, transcripts, CDS, proteins); (3) Search: to find genes by their identifiers or annotation keywords; (4) Genome browser: to explore the genome and its gene models in a genomic context, and visualize gene expression data coverage of the ‘Picual’ experiments available in OliveAtlas (Figure S10); (5) Sequence extraction: to get the sequences from a list of genes (genome, transcripts, CDS, proteins), and (6) Annotation extraction: to get the annotations from a list of genes (TrEMBL, SwissProt, TAIR10, InterPro).

#### 2.5.5. Expression Viewer Use Case Example

Six differentially expressed genes from the “Picual stress” dataset [18] were selected because they showed different responses, in leaves and roots, to cold stress, infection with *V. dahliae,* and wounds in roots (Table 4). As a first step, the gene list was pasted in the “Annotation Extraction” tool in OliveTreeDB, to obtain the putative annotation of these genes based on their homology with proteins from the TAIR and UniProt databases and the protein domains identified with InterProScan. There, we could download the resulting table in spreadsheet format, which includes the putative annotations that will help us to interpret the function of the genes in the stress conditions in which they are expressed.

Then, the six gene names were pasted in the “Expression Viewer” input form, after selecting the “Picual stresses” dataset, to know their expression. When comparing the expression of the selected genes in the “Lines” plot (Figure 4), it was observed that four of the genes, in orange, pink, red and yellow, were highly expressed in leaves in response to 24 h of cold, clearly with higher expression than in control conditions. Two of these genes were annotated as arginine decarboxylases, and the other two as stachyose synthase, and dehydration-responsive element-binding protein, respectively. The gene Oleur061Scf3147g04027, in dark yellow and annotated as fatty acid hydroxylase, showed a high and differential expression in roots in response to *V. dahliae* infection after 15 days. The gene Oleur061Scf0768g00001, in green, and annotated as FAD-binding Berberine-like, showed a high and differential expression in roots in response to *V. dahliae* infection after 48 h, and a high expression in roots in response to wounds, especially in a short term, with a peak in the 24 h.

In the “Expression cards” (Figure 5), using Oleur061Scf2532g00003 as an example, the gold card highlighted the highest expression of the gene in leaves in response to cold after 24 h, showing a photograph of the plant in those conditions. This gene was also expressed in leaves in control conditions but showed almost no expression in the rest of the experimental conditions. Five black cards show where the gene was not expressed, and 6 white cards showed experiments with an expression below 1 TPM. Photographs display the phenotype of the plant in each experimental condition, and cartoons represent the experimental conditions when no photos are available.

In the “Heatmap” visualization (Figure 6), as in the “Lines” plot (Figure 4), we can observe that four genes were highly expressed in leaves exposed to low temperature for 24 h. Oleur061Scf0768g00001 showed high and differential expression values in root wounding and infection with *V. dahliae*, especially at 48 and 24 h, respectively. Additionally, Oleur061Scf3147g04027 showed high expression in roots infected with *V. dahliae* at 15 days.

In the “Replicates” plot (Figure 7), for the selected gene Oleur061Scf3147g04027, we can observe the two biological replicates of each experiment showed very similar expression values, which made them hard to be differentiated in the graph. Only “Roots *Verticillium* 15d” shows two points clearly differentiable but very close to each other. In other cases, this visualization might allow the identification of unexpected behaviors between replicates.

Finally, we can explore the expression of each sample in the “Average values” table (Figure 8). Here, we could copy the table in the clipboard or download it to combine it with the previous spreadsheet including the gene annotations (obtained using the OliveTreeDB “Annotation Extraction” tool). That way, it was possible to combine expression and annotation data of genes of interest in a simple and comfortable format to work with.

## 3. Discussion

Different analyses to cluster the experiments of OliveAtlas show a clear distribution and separation of the samples by tissue type and experimental condition, standing out pollen samples as more different from the rest. Pollen samples had a lower number of expressed genes than the rest of the tissues and they showed a much higher number and proportion of specific genes. This is well known and has been described before in other plants such as maize [24] and Arabidopsis [25]. On the other hand, roots were the organ with the most expressed and specific genes, many of them were involved in morphogenesis, which might be explained due to the 4-month-old root samples of young plants actively growing. The stem, with only 193 genes, had the lowest number of specific genes in comparison with all the other tissues. This could be expected since many of the genes expressed in stems might also be expressed in other tissues involved in development or photosynthesis.

Regarding the biological functions enriched in specific genes from seeds, leaves, pollen, and roots in control conditions, most of the terms found are expected according to the processes occurring in these organs. In the seeds, terms related to metabolic and biosynthetic processes, development, chromosome organization, and organ morphogenesis are expected in a tissue containing the embryo. Some terms related to floral development could be explained by common functions in development that are assigned to Gene Ontology terms of genes expressed in flowers, but also expressed in other tissues. In leaves, is completely normal to find terms related to photosynthesis and energy. However, both leaves and roots had defense response terms, which could be explained by samples from adult individuals planted on fields that could be exposed to multiple stresses. The term related to response to wounding could be due to the sample collection or exposure to the natural environment. Roots also showed expected terms related to morphogenesis and metabolic processes. Ultimately, pollen germination samples showed biological processes related to pollen such as pollen tube growth, morphogenesis, and vesicle and ion transport.

On the other hand, considering the 26 ‘Picual’ RNA-seq experiments in OliveAtlas, which represent a wide number of tissues and experimental conditions, around 10% of the genes (8319 genes) showed no expression, and 30% of them (25,136) showed an expression value below 2 TPM. This indicates that more experiments are still needed to confirm the expression of some of these genes and might also indicate the cv. Picual gene model annotation is overestimated. It is important to note that a recent study [9] showed that the ‘Picual’ assembly had similar BUSCO completeness values but twice the percentage of duplicated genes compared to the ‘Farga’ genome. The high number of duplicated genes in ‘Picual’ could suggest the presence of some artefactual duplications, probably due to high levels of heterozygosity in some regions of the genome, which could confound the assembler to collapse the haplotypes correctly. The resources included in OliveAtlas could be of great utility for future improvement of the ‘Picual’ gene model annotation.

In the “Expression Viewer” use case example, one gene annotated as arginine decarboxylases, two as stachyose synthase, and another one as dehydration-responsive element-binding protein, showed a high and differential expression in response to 24 h of cold treatment. The arginine decarboxylase has been described as a key gene in the response to cold by promoting putrescine biosynthesis [26,27], which modulates ABA biosynthesis to activate the expression of cold-responsive genes [28,29]. Stachyose plays an important role in conferring desiccation tolerance [30] and stachyose synthase has been described to be induced under cold stress in many other studies [31,32,33]. The dehydration-responsive element-binding protein belongs to the AP2/ERF transcription factor family and is known to be induced in low temperatures to promote freezing tolerance in plants [34,35]. Moreover, it could be an activator of arginine decarboxylase [27], one of the genes that also showed a high expression in this sample.

Oleur061Scf3147g04027 showed a high and differential expression in roots in response to *V. dahliae* infection after 15 days. The annotations of this gene in OliveTreeDB show it is a homologous gene to *CER1*, also known as ECERIFERUM 1, with uncharacterized Wax2 C and fatty acid hydroxylase domains. This gene has a key role in wax biosynthesis, which forms a hydrophobic external layer that protects the plant by limiting the attachment of bacteria and fungi [36].

The gene annotated as FAD-binding Berberine-like showed a high and differential expression in roots in response to *V. dahliae* infection after 48 h, and a high expression in roots in response to wounds, especially in a short term, with a peak in the 24 h. The FAD-binding Berberine-like genes are related to cell wall biosynthesis and might be involved in oligogalacturonides homeostasis, which accumulates in the extracellular matrix after wounding and has been described to participate in the defense against fungi [37,38].

OliveAtlas, together with OliveTreeDB, is a useful platform for olive research, providing multiple bioinformatics tools to explore gene expression data, which are essential for experimental design and interpretation of results. In the future, it is expected that the olive research community will analyze gene expression data based on the available olive genome references, which will allow the expansion of OliveAtlas datasets, including expression data from more varieties and experimental conditions. We encourage researchers to contact us for the addition of new datasets or the submission of their results in NCBI GEO for simple inclusion in OliveAtlas. Our system, based on easyGDB, only requires adding a tab-delimited file with the normalized expression values of the replicates (as in the OliveAtlas downloads section) to be automatically available in the gene expression tools. The gene IDs can be linked to any of the available olive genomic references in Phytozome (Wild olive), OliveTreeDB (‘Picual’), CNAG olive genome page (‘Farga’ Oe9), Ensembl Plants, or any other site. Additionally, more bioinformatics tools such as a gene co-expression tool are planned to be developed.

## 4. Materials and Methods

### 4.1. RNA-Seq Data Downloading

Expression data from seven of the nine RNA-seq data sets available in OliveAtlas were published by Leyva-Pérez et al. [18] and by Ramírez-Tejero et al. [17,20]. The other two are in preparation for publication [19]. All RNA-seq experiments of *O. europaea* cv. Picual available at the Sequence Read Archive (SRA) were identified and the ones with replicated experiments were selected. Datasets were obtained from BioProjects PRJNA590386, PRJNA256033, and PRJNA638671, and RNA-seq data from seed (in preparation, PRJEB59252) and pollen tissues (in preparation, PRJEB59024) are unpublished data generated by the research groups involved in OliveAtlas development. The SRA Toolkit v.2.11.2 (https://trace.ncbi.nlm.nih.gov/Traces/sra/sra.cgi?view=software (accessed on 24 February 2023)) was used to download original files in the SRA format with the prefetch program. Therefore, SRA files were converted to compressed FASTQ files using fasterq-dump.

### 4.2. Data Processing and Normalization

All the FASTQ files were preprocessed using fastp v.0.22.0 [39], with the options -f 12, -q 20, and -l 30, to remove adapter sequences and low-quality reads. The clean sequences were mapped to the *Olea europaea* cv. Picual genome v061 [12] using HISAT2 v.2.1.0 [40], and were converted to BAM and sorted with Samtools v.1.13 [41]. FeatureCounts [42] included in the Subread v.2.0.3 package [43] was used for quantification. Gene counts were normalized to Transcripts per Million (TPM) using the R function convertCounts from the package DGEobj.utils, and were used for the datasets presented in OliveAtlas, for the identification of specific genes, and for the functional enrichment. On the other hand, for the PCA and the calculation of the coefficient of variation (CV), gene counts were filtered by a minimum of 1 CPM (Counts Per Million Mapped) in 1 library and then normalized to Trimmed Mean of M-values (TMM) using edgeR v.3.38.2 [44]. The data from the experiments of multiple varieties with different tolerance to *Verticillium* [20] and for the drought experiments in cv. Souri [21] were downloaded from Gene Expression Omnibus (GEO) and included in OliveAtlas as in the original publication [20]. The ‘Souri’ dataset was converted from normalized counts to TPM.

### 4.3. Gene Clustering and Enrichment Analyses

Principal component analysis (PCA), specific gene identification, CV calculation, and enrichment analyses were done in R v.4.2.1. The experiment replicates were clustered in a PCA plot using logarithmic values in the prcomp function included in stats v.4.2.1. Specific genes were identified for each tissue in control conditions based on a minimum value of TPM ≥ 2, and their functional enrichment was analyzed with clusterProfiler v.4.4.4 package [45] using the most similar protein in the *Arabidopsis thaliana* TAIR10 protein set. The wordcloud package v2.6 was used for word cloud representation. The coefficient of variation was calculated as in [46] to identify the least variable genes with a CV < 0.2.

### 4.4. Gene Lookup Dataset Calculation

Most similar proteins between ‘Picual’ and the other available genomes, ‘Farga’, ‘Arbequina’ and wild olive (*O. europaea* subsp. *sylvestris*), were identified using Diamond BLASTp v.2.0.14 [47] with the option —very-sensitive and —max-target-seqs 1. The same method was used to identify the most similar proteins between ‘Picual’ and *A. thaliana*.

Most similar proteins between ‘Picual’ and the four transcriptome assemblies available at ReprOlive, pollen, pistil, vegetative tissues, and seed tissues, were identified with Diamond BLASTx v.2.0.14 with similar parameters.

### 4.5. Expression Atlas Implementation

OliveAtlas is based on EasyGDB [22], and it can be accessed at https://www.oliveatlas.uma.es/ (accessed on 24 February 2023). The code used to customize OliveAtlas is available on GitHub (https://github.com/noefp/olive_atlas (accessed on 24 February 2023)).

## Figures and Tables

**Table 1:** Available expression datasets in OliveAtlas.

Dataset Name	Number of Experiments	Publications
Picual all tissues ^1^	10	[17,18,19]
Picual plant organs	6	[17]
Picual pollen germination	4	[19]
Picual seed tissues	3	
Picual stresses	13	[18]
Picual cold stress ^2^	3	[18]
Picual wound stress ^2^	6	[18]
Picual *Verticillium* infection ^2^	6	[18]
Roots of olive cultivars with variable tolerance to *Verticillium*	36	[20]
Souri drought ^3^	8	[21]

^1^ Dataset grouping all different tissue samples from cv. Picual in control conditions. ^2^ Datasets for responses of leaves and roots to cold, wound, and *Verticillium dahliae* infection, respectively, formed by experiments from the ‘Picual’ stresses dataset. ^3^ Based on the wild olive genome reference.

**Table 2:** Summary of the number of genes expressed in each ‘Picual’ tissue/organ.

Tissue/Organ	# Expressed Genes	# Specific Genes	% Specific Genes
Roots	37,707	2261	6.00
Meristems	37,022	999	2.70
Flowers	35,023	1914	5.46
Stems	34,154	193	0.57
Leaves	33,806	608	1.80
Fruits	30,992	483	1.56
Seeds	26,484	832	3.14
Pollen	13,380	1469	10.98

**Table 3:** Expression and coefficient of variation of least variable genes.

Gene ID	Functional Annotation	TPMs (Mean)	CV
Oleur061Scf3144g02017	Probable serine/threonine protein kinase PBL8	41.53	0.1681
Oleur061Scf8008g02017	Casein kinase 1-like protein	166.25	0.1808
Oleur061Scf1177g07035	Serine/arginine-rich splicing factor SR45a-like	50.87	0.1845
Oleur061Scf1733g08033	Putative MO25-like protein At5g47540	43.07	0.1906
Oleur061Scf3049g03001	FRIGIDA-like protein	21.85	0.1920
Oleur061Scf6724g01003	Histone acetyltransferase of the MIST family 1-like	45.89	0.1983
Oleur061Scf9161g10012	Vacuolar protein sorting-associated 20 homo-log 2-like	24.52	0.1994

**Table 4:** Use case example genes and their putative annotations.

Gene ID	Gene Annotation
Oleur061Scf0768g00001	FAD-binding Berberine-like
Oleur061Scf2532g00003	Dehydration-responsive element-binding protein
Oleur061Scf3147g04027	Fatty acid hydroxylase
Oleur061Scf3552g08038	Arginine decarboxylase
Oleur061Scf7114g00004	Arginine decarboxylase
Oleur061Scf8024g00001	Stachyose synthase

## Data Availability

Raw data of the OliveAtlas datasets are available in BioProjects PRJNA590386, PRJNA256033, PRJNA638671, PRJEB59252, PRJEB59024, and PRJNA606032. The gene expression data of endosperm and embryo samples will be released after publication. The gene expression atlas tool is available at https://www.oliveatlas.uma.es/ (accessed on 24 February 2023) and the code used to customize OliveAtlas is available in GitHub (https://github.com/noefp/olive_atlas (accessed on 24 February 2023)).

## Supplementary Materials

The following supporting information can be downloaded at: https://www.mdpi.com/article/10.3390/plants12061274/s1, Table S1: specific genes; Table S2: specific genes venn; Table S3: enrichment specific genes venn; Figure S1: OliveAtlas home page; Figure S2: OliveAtlas about page; Figure S3: OliveAtlas downloads; Figure S4: OliveAtlas dataset description; Figure S5: OliveAtlas gene expression atlas input; Figure S6: OliveAtlas expression comparator; Figure S7: OliveAtlas gene lookup tool; Figure S8: OliveAtlas gene set enrichment tool; Figure S9: OliveAtlas links to OliveTreeDB tools; Figure S10: OliveTreeDB genome browser.
