# Supplementary material for: OliveAtlas: A Gene Expression Atlas Tool for Olea europaea
Source: Plants (Basel). 2023 Mar 10;12(6):1274. doi: 10.3390/plants12061274 (PMC10053119; doi:10.3390/plants12061274)
Supplement: Supplementary file 1 [file plants-12-01274-s001.zip › OliveAtlas - Supplementary figures.pdf]

OliveAtlas: A gene expression atlas tool for *Olea europaea*

Supplementary material.

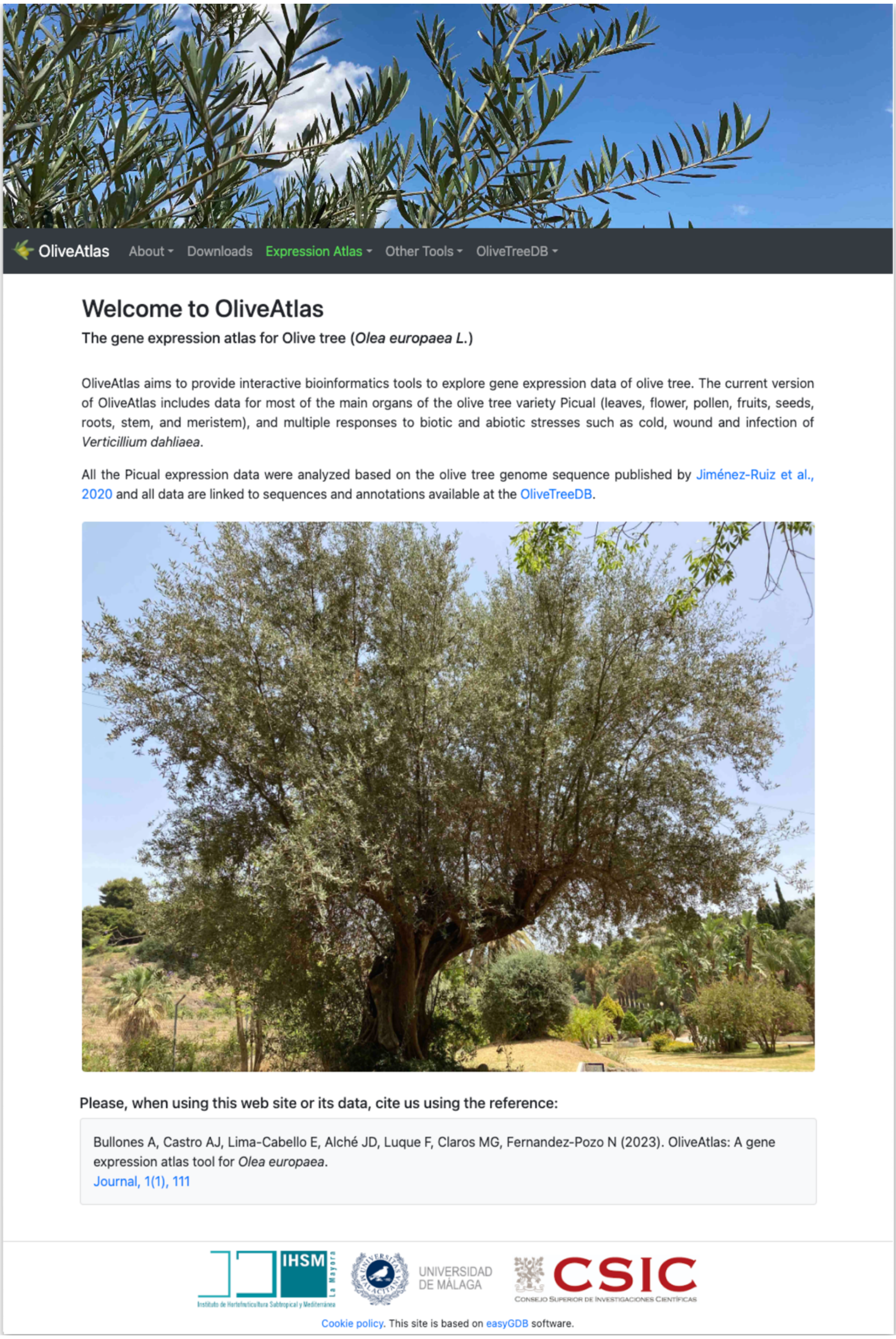

Figure S1. OliveAtlas home page.

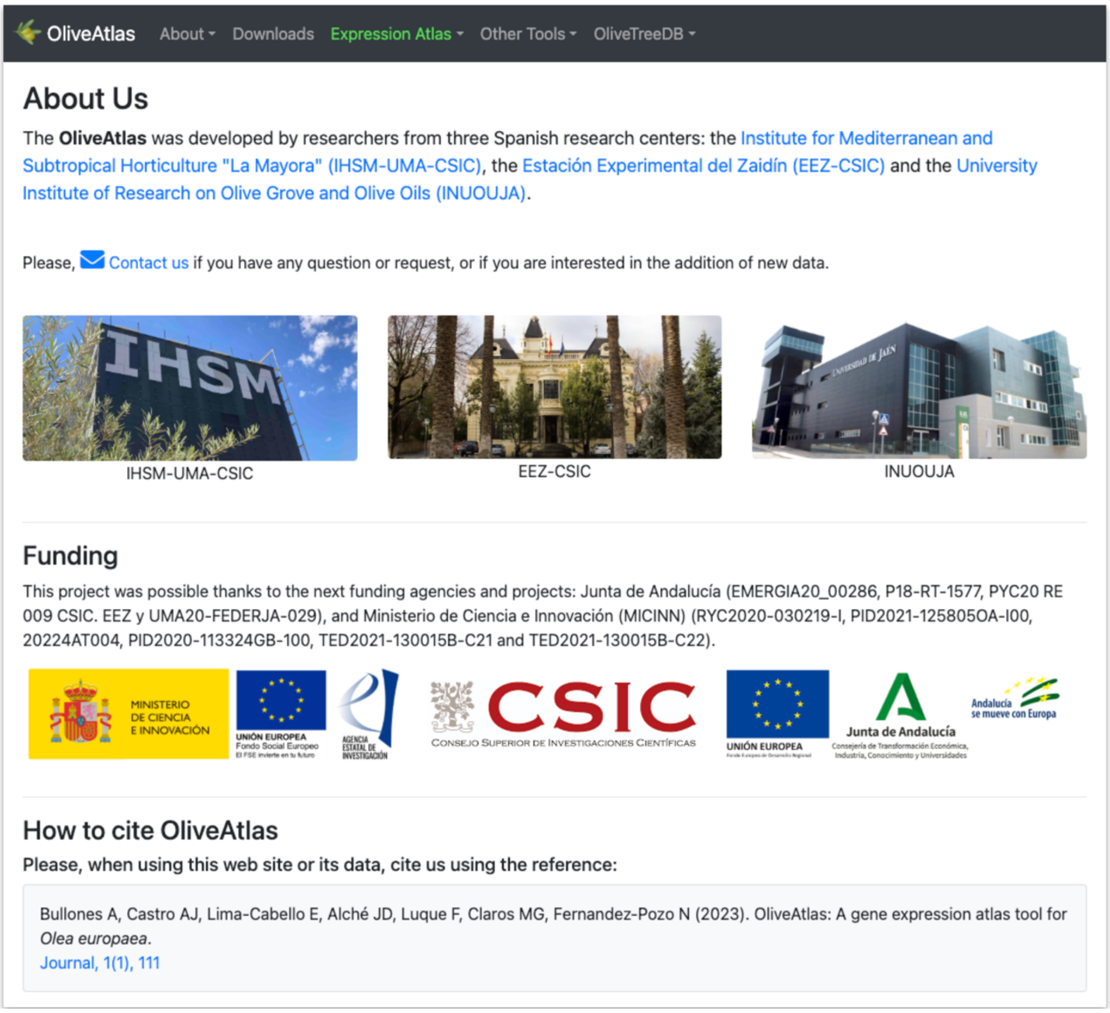

Figure S2. OliveAtlas about page. It contains information about the institutions involved in the project, funding, and how to cite the related publications.

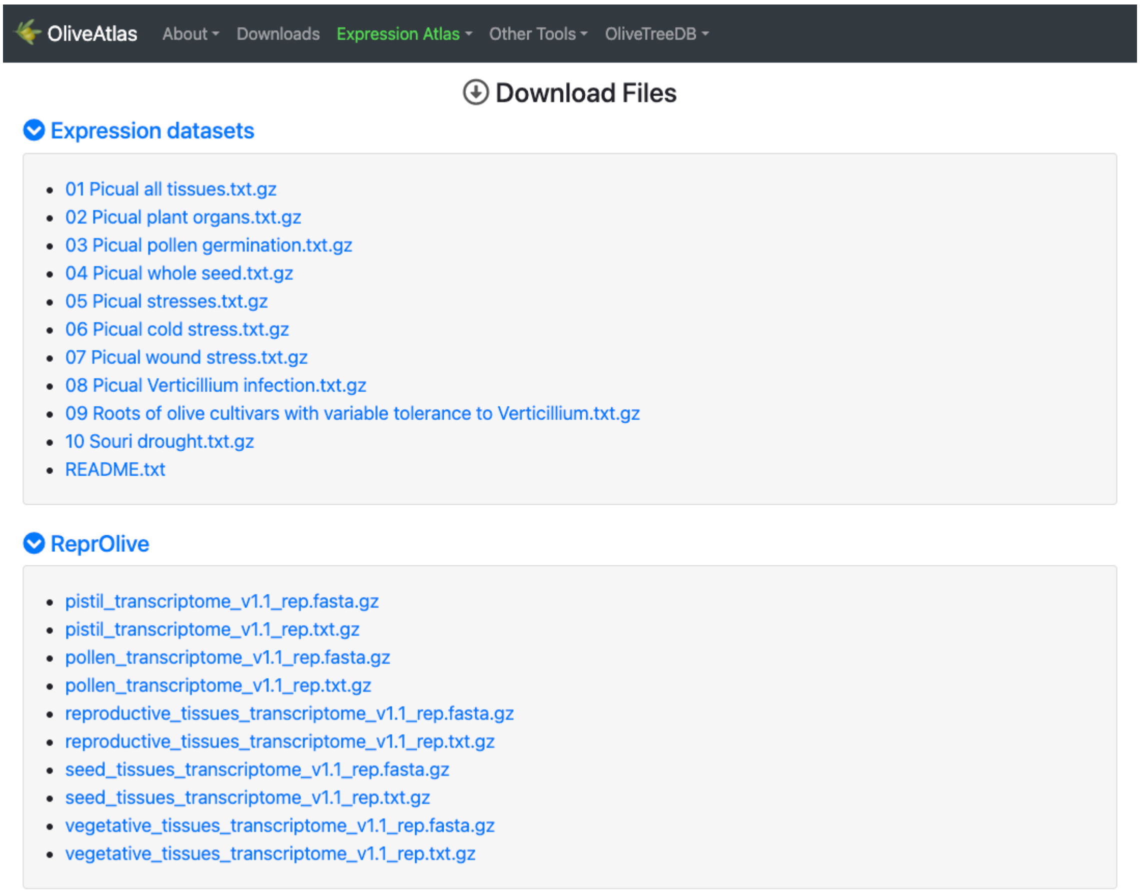

Figure S3. OliveAtlas downloads page.

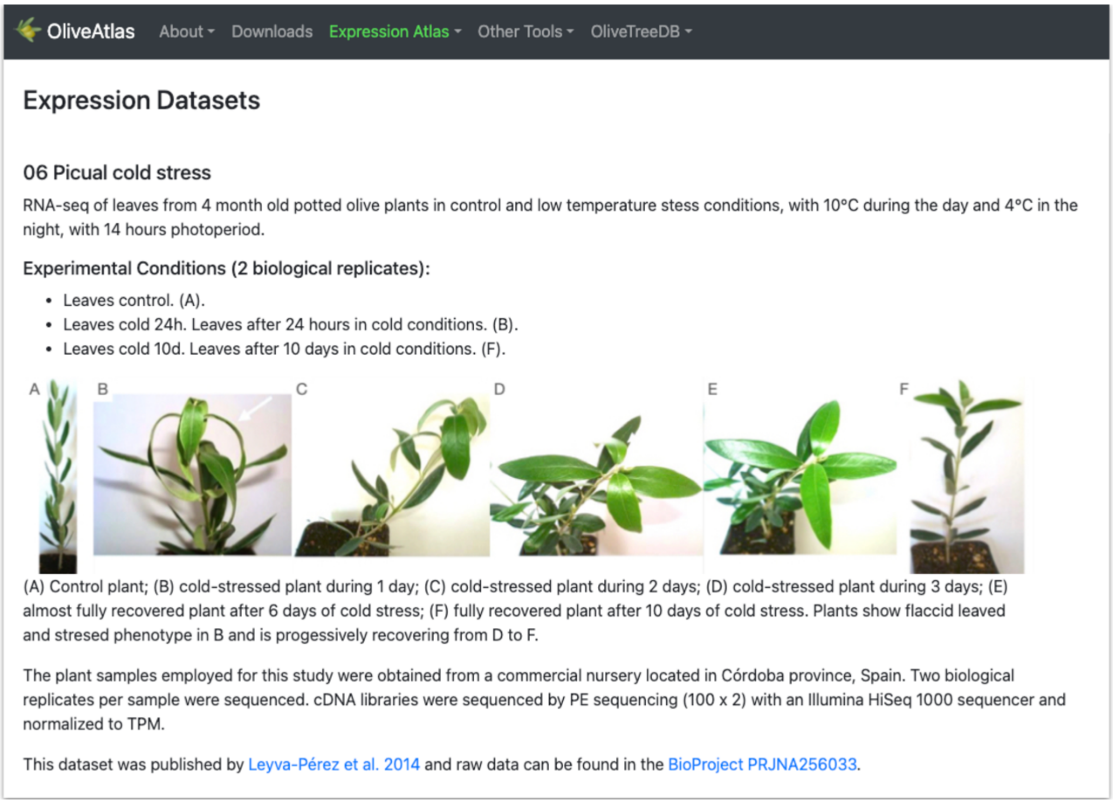

Figure S4. OliveAtlas dataset description page.

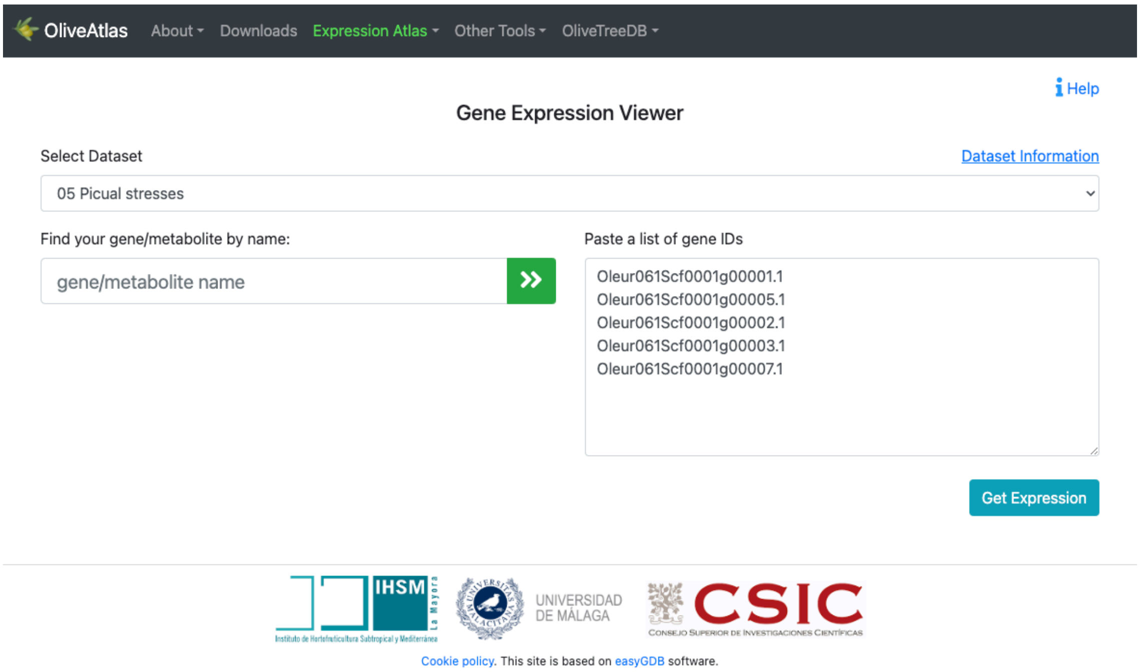

Figure S5. OliveAtlas "Expression Viewer" input menu. It includes dataset selection, gene name autocompletion, datasets information link and the possibility of pasting one or multiple gene names as input.

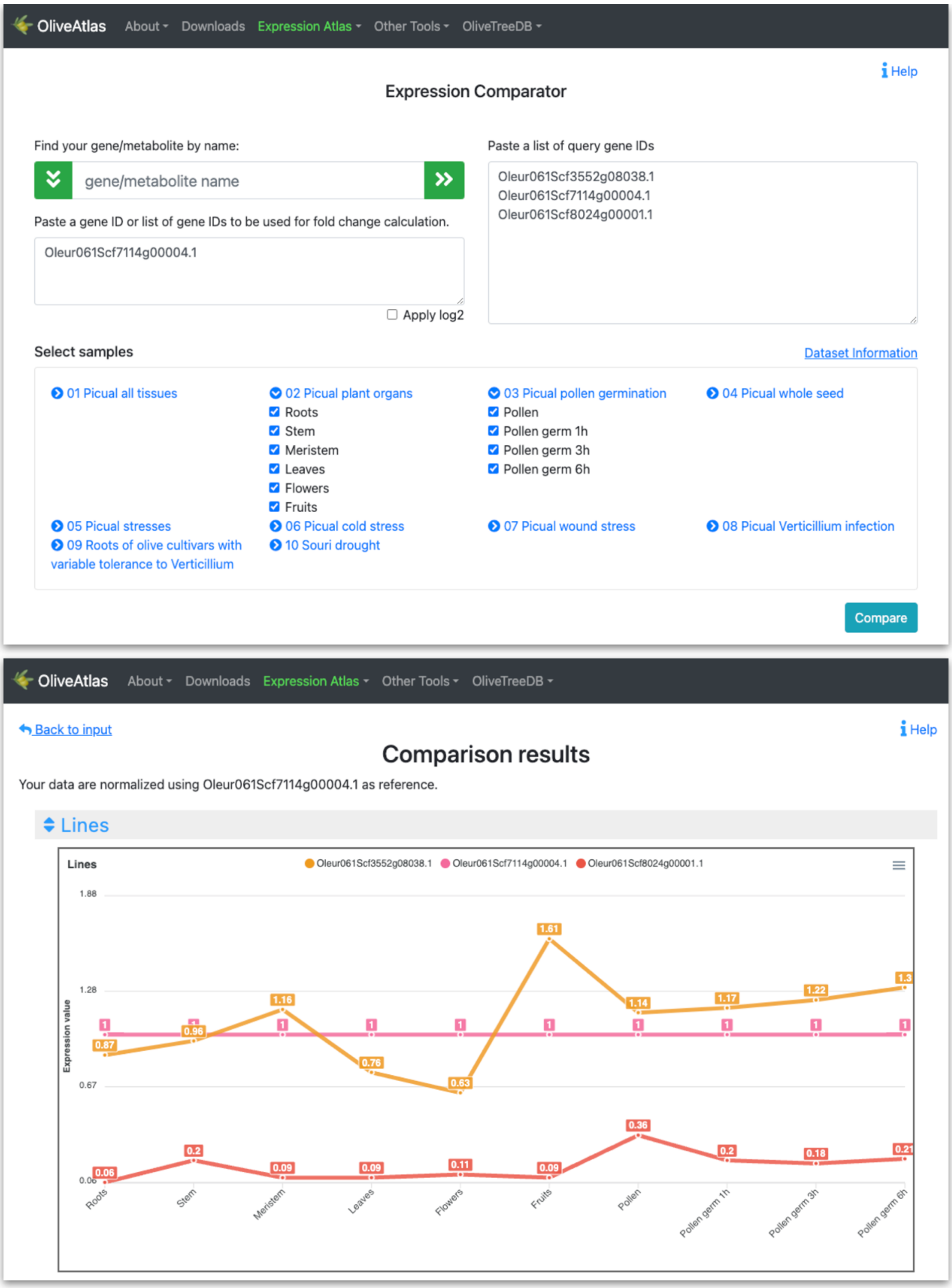

Figure S6. OliveAtlas "Expression Comparator". Input page, on top, includes sample selection, gene name autocompletion, and the possibility of normalizing by relative expression calculating fold-change or log-ratios. Output page, at the bottom, shows the fold-change values of three genes in the "Lines" plot.

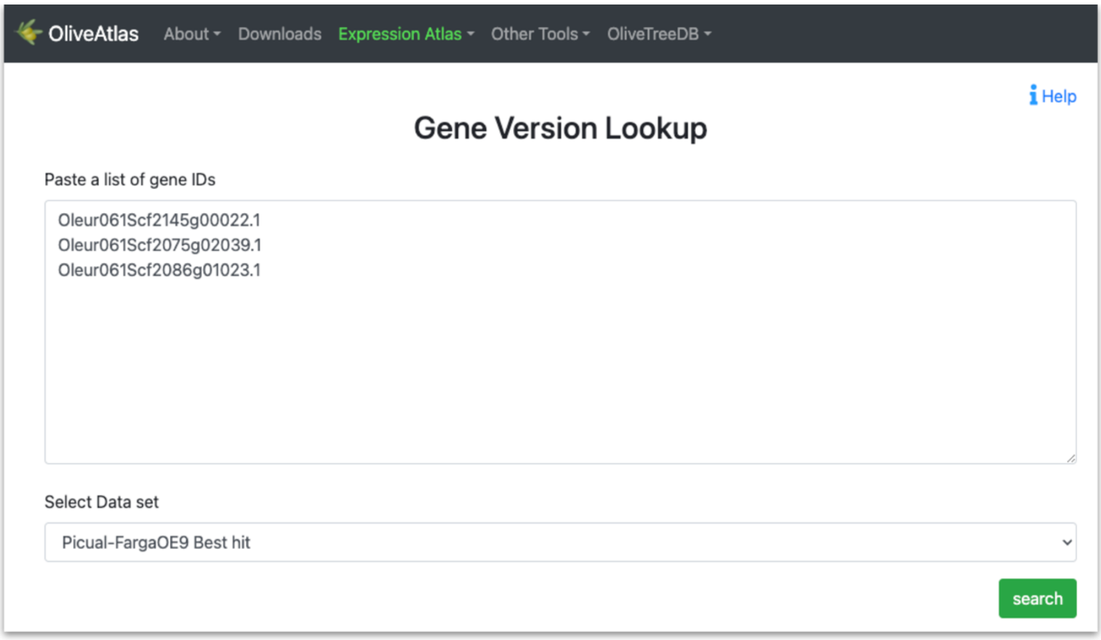

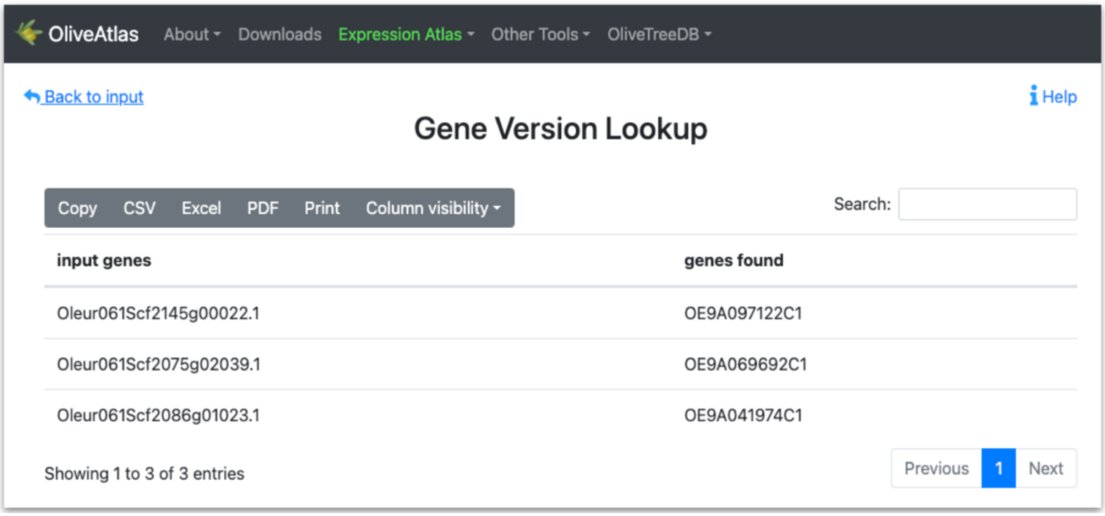

Figure S7. OliveAtlas "Gene lookup" tool. Input page on top and results page at the bottom.

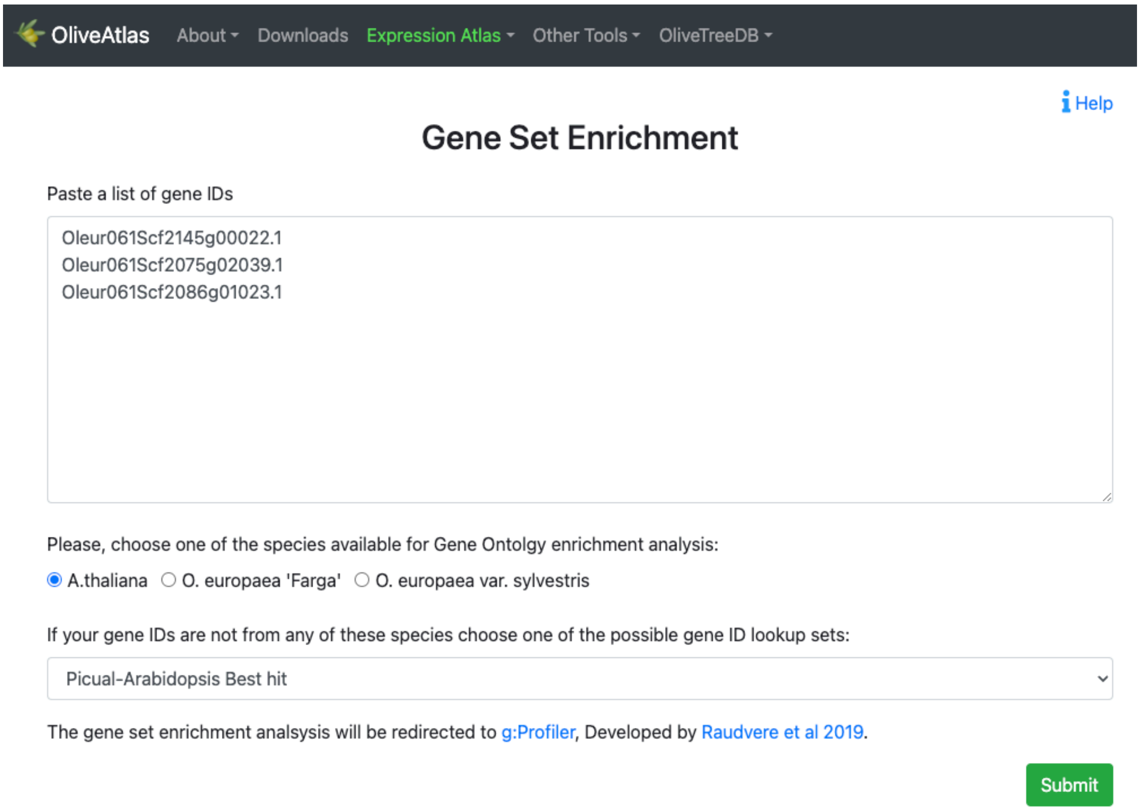

Figure S8. OliveAtlas "Gene Set Enrichment" tool input page.

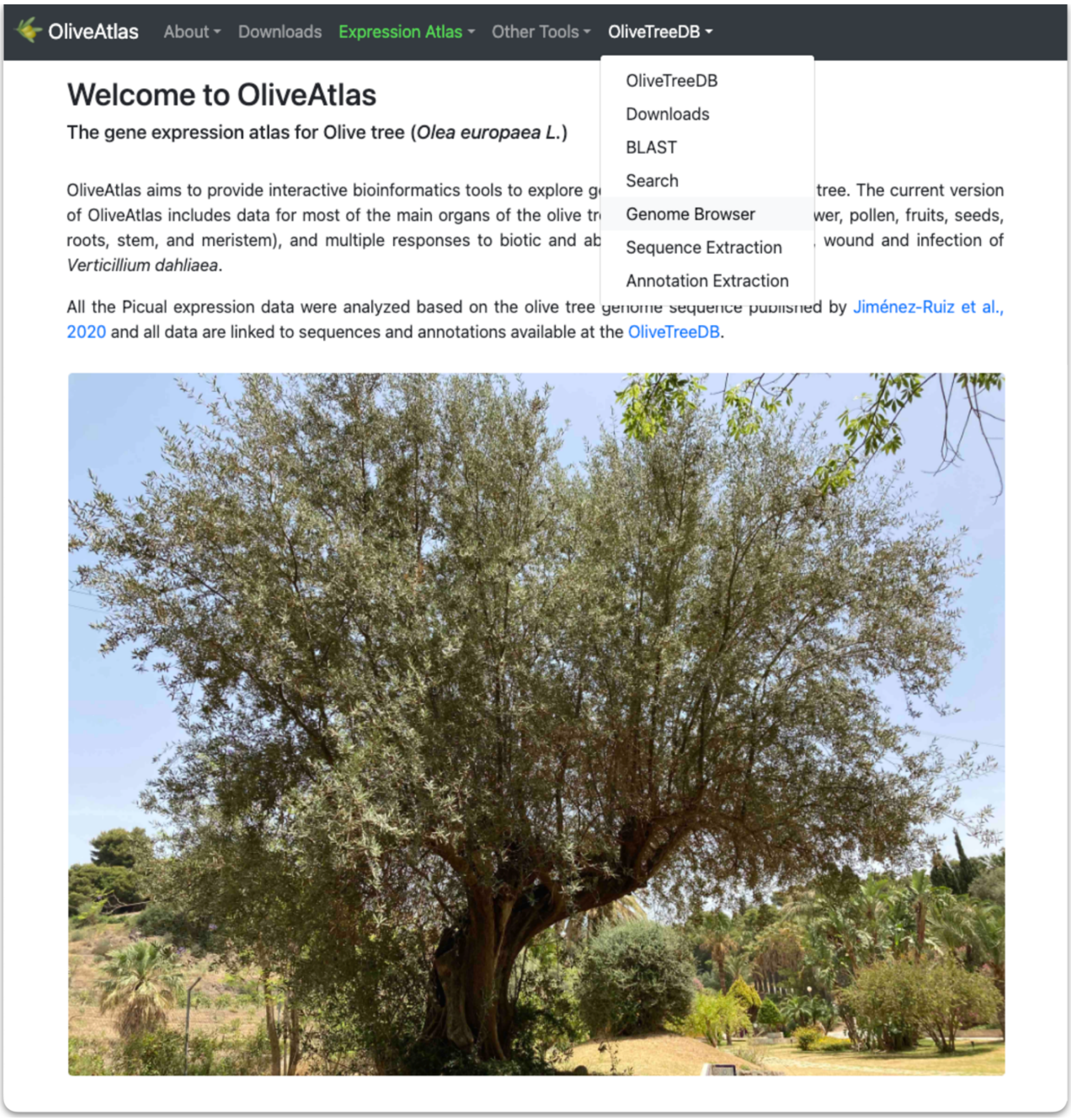

Figure S9. OliveAtlas links to the OliveTreeDB data and tools. The dropdown menu "OliveTreeDB" lists the available links to the 'Picual' genomic portal: (1) OliveTreeDB, including (2) Downloads, (3) BLAST, (4) Search, (5) Genome Browser, (6) Sequence Extraction, and (7) Annotation Extraction.

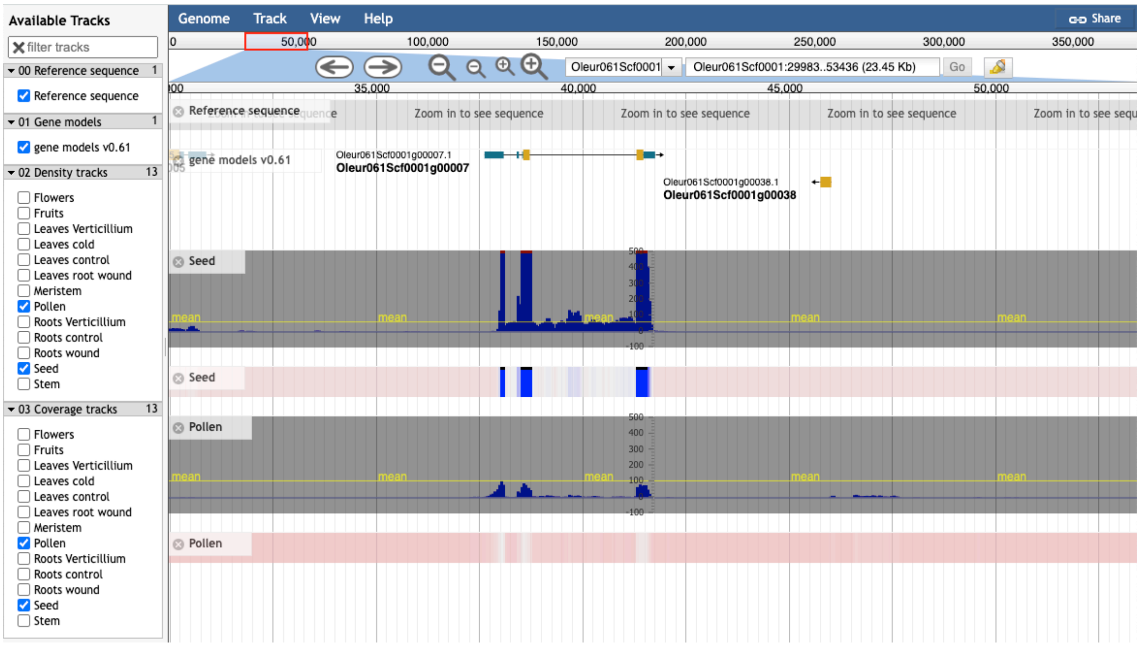

Figure S10. Genome browser in OliveTreeDB, including density and coverage tracks of the expression experiments from OliveAtlas.
